# The Relation of Sciatica to Organic Stricture of the Urethra

**Published:** 1888-02

**Authors:** J. W. Griggs

**Affiliations:** West Point, Ga.


### THE RELATION OF SCIATICA TO ORGANIC STRICTURE OF THE URETHRA.*

BY J. W. GRIGGS, M. D., WEST POINT, GA.

I disclaim any intention whatever of creating the impression that I do not believe in the existence of sciatica proper, irrespective of the above heading; but I do enter my protest against the indiscriminate use of the term when applied to every pain in the hip, however remote the cause. I am well assured that nine cases in ten of the so-called sciatica are simply nervous reflexes incident to organic stricture of the urethra. In submitting my cases to the consideration of the Association, I do so in a measure to direct the attention of the profession to a subject new in theory at least, if not in practical demonstration.

September, 1886, I attended Captain W. L. W. during an attack of what seemed at the time to be a most severe form of sciatica. I instituted the usual remedies in that morbid condition of the nervous system which, of course, were based upon general principles, even to the administration of electricity, and the application of a large blister to the sacrum, and in five or six weeks I could not discern the slightest improvement in the condition of my patient. About two weeks later the pain became less severe; appetite returned, and he gradually began to move about the house with the aid of crutches, though it was sometime before he was fully restored to his normal condition of health. Toward the latter part of December, 1886, he approached me upon the subject of the return of his sciatica (?). He complained greatly of pain in the back and hip, and spoke of the great annoyance in being unable to sit with any degree of comfort in his chair. His pain grew worse daily, and it was most mortifying to see the sufferer losing spirit and growing weaker day by day. Knowing that nothing I had done for him had been more than temporary,

*Read before the Medical Association of Georgia. From the Transactions.

and he was in a most pitiable state; knowing that an operation had been performed upon him in 1876, by Dr. W. F. Westmoreland, for organic stricture, in which perineal section had first to be made, I determined to inquire of him whether the present suffering could have any reference to the former case or not. He was positive in affirming that he felt nothing of his old stricture, and that I was mistaken in my suspicions. In the afternoon of the day on which this conversation took place, my patient informed me that he, for the first time, had felt the want of power to concentrate his mind on the act of micturition; that he seemed to have lost power over the accelerator muscles, as I understood it, as well as the rectum in defecation. I at once gave him a small quantity of chloroform, and endeavored to insert a No. 10 silver catheter in the bladder, but it was firmly grasped as it entered the prostatic portion of the canal, and all methods known to me failed to effect an entrance. I managed to draw the urine through a No. 4 gum catheter. I then made my preparations as well and as fast as possible for breaking down the obstruction, deeming it the whole source of the trouble. The operation was only partially successful. As I was performing divulsion (with Thompson's divulsor) the cross-bar broke just as the blades registered No. 2. I quickly removed the instrument, and immediately inserted a No. 16 French bougie. Gave patient a hot hip bath, put him to bed, and inserted 1/3 gr. sulph. morphia hypodermically. He rested well all night. Missed the much-dreaded chill which so often follows divulsion. Pain subsided and appetite returned. Within a week he was out looking after his business. And though he only inserts a No. 13 French sound, with some little difficulty, he experiences no more of the disagreeable symptoms of sciatica.

I have still another case of similar history to present. Mr. J. E. H., a young gentleman, had previous history of organic stricture which had been divulsed in 1881. January, 1887, he consulted me in regard to what seemed to be sciatica, but my recent experience had taught me to look with distrust upon sciatica (?). So I began a critical examination into the cause of the pains complained of, and upon sounding found the stricture hard and resisting. Divulsed under chloroform, and patient soon regained his former spirits and health. He is able to use a No. 32 steel sound, and, with only slight pain in the left testicle, is well.

A short time ago Mr. O. D. W. consulted me concerning his general health. Among other things he complained of a lack of vitality, and incidentally mentioned a severe and continuous pain in the back and hip. I found, on examination, that he had an organic and spasmodic stricture. I treated him by gradual dilatation, and while he is not entirely well of the stricture, he says that he suffers no pains at all, and the impotency is perfectly overcome.

I was asked to examine a child, in the winter, for what was supposed to be hip-joint disease. The child seemed, at a casual glance, to be suffering from *morbus coxarius*, but a closer examination revealed an adherent prepuce with highly irritable glands and some ulceration. Circumcision was performed, and in the course of one month all deformity and ill health had disappeared.

Hastily throwing together these few cases of morbid reflexes, I herein express the hope that others of the profession will come forward and assist in the effort to separate a sciatica, so-called, from organic stricture of the urethra.

